# Degree of protection provided by poverty alleviation policies for the middle-aged and older in China: evaluation of effectiveness of medical insurance system tools and vulnerable target recognition

**DOI:** 10.1186/s12961-022-00929-9

**Published:** 2022-11-14

**Authors:** Wanxin Tian, Bing Wu, Yahong Yang, Yongqiang Lai, Wenqing Miao, Xiyu Zhang, Chenxi Zhang, Qi Xia, Linghan Shan, Huiying Yang, Huiqi Yang, Zhipeng Huang, Yuze Li, Yiyun Zhang, Fan Ding, Yulu Tian, Hongyu Li, Xinwei Liu, Ye Li, Qunhong Wu

**Affiliations:** 1 Research Center of Public Policy and Management, School of Health Management, Harbin Medical University, No. 157 Baojian Road, Nangang District, Harbin, 150086 Heilongjiang China; 2 The Second Affiliated Hospital of Harbin Medical University, Harbin Medical University, Harbin, Heilongjiang China; 3 Department of Medicine, Jiamusi University, Jiamusi, 154007 Heilongjiang China; 4 School of Ethnology and Sociology, Yunnan University, Kunming, Yunnan China; 5 Nursing Department, Heilongjiang Provincial Hospital, Harbin, Heilongjiang China; 6 Department of Epidemiology and Health Statistics, School of Public Health and Management, Ningxia Medical University, Yinchuan, Ningxia China

**Keywords:** Multi-vulnerability, Middle-aged and older people, Medical insurance, Poverty reduction, Catastrophic health expenditure (CHE)

## Abstract

**Background:**

China’s medical insurance schemes and poverty alleviation policy at this stage have achieved population-wide coverage and the system's universal function. At the late stage of the elimination of absolute poverty task, how to further exert the poverty alleviation function of the medical insurance schemes has become an important agenda for targeted poverty alleviation. To analyse the risk of catastrophic health expenditure (CHE) occurrence in middle-aged and older adults with vulnerability characteristics from the perspectives of social, regional, disease, health service utilization and medical insurance schemes.

**Methods:**

We used data from the 2018 China Health and Retirement Longitudinal Study (CHARLS) database and came up with 9190 samples. The method for calculating the CHE was adopted from WHO. Logistic regression was used to determine the different characteristics of middle-aged and older adults with a high probability of incurring CHE.

**Results:**

The overall regional poverty rate and incidence of CHE were similar in the east, central and west, but with significant differences among provinces. The population insured by the urban and rural integrated medical insurance (URRMI) had the highest incidence of CHE (21.17%) and health expenditure burden (22.77%) among the insured population. Integration of Medicare as a medical insurance scheme with broader benefit coverage did not have a significant effect on the incidence of CHE in middle-aged and older people with vulnerability characteristics.

**Conclusions:**

Based on the perspective of Medicare improvement, we conducted an in-depth exploration of the synergistic effect of medical insurance and the poverty alleviation system in reducing poverty, and we hope that through comprehensive strategic adjustments and multidimensional system cooperation, we can lift the vulnerable middle-aged and older adults out of poverty.

**Supplementary Information:**

The online version contains supplementary material available at 10.1186/s12961-022-00929-9.

## What is known on this topic

To our knowledge, most existing studies use past data to analyse the factors that may contribute to catastrophic health expenditures (CHE). Although there are studies demonstrating the impact of economic level on the incidence of CHE in a particular province or more broadly in eastern, central and western China, more in-depth inter-province comparisons are lacking. And most research on CHE is specific to rural populations, focusing on the New Rural Cooperative Medical Scheme (NRCMS).

What this study adds:

Our research is time-sensitive and complements the above-mentioned gaps. In addition, we innovate by linking poverty alleviation policies to medical insurance in China to achieve synergistic support for the middle-aged and older groups who are vulnerable to health poverty. Following are the key findings of this research:

- Interregional macroeconomic development is no longer the main force in reducing the regional incidence of CHE.
- Basic health insurance does not provide stronger financial protection for the middle-aged and older adults.
- Urban and rural integrated medical insurance (URRMI) should not be limited to simple integration, but should also enhance benefits for vulnerable middle-aged and older people.

## Background

In China, poverty alleviation has always been an important issue for the national economy and people's livelihoods. In 1990, China's poverty rate was over 60%, and with the tireless efforts of Chinese governments, it dropped to 1.7% in 2018. In 2020, China eliminated absolute poverty [1, 2]. As an important module in China's poverty alleviation process, basic medical insurance has played a huge role in the process of comprehensively promoting poverty alleviation. Moreover, the construction process for China's medical insurance schemes and the poverty alleviation policy are complementary to each other. From reform and opening up in 1978 to 2012, China's poverty alleviation process underwent three stages, namely institutional reform, large-scale development and action at the village level, which lifted more than 600 million people out of poverty [3]. During this process, China's medical insurance schemes were also established one after another. In 1998, 2003 and 2007, the Urban Employee Basic Medical Insurance (UEBMI), the New Rural Cooperative Medical Scheme (NRCMS) and the Urban Resident Basic Medical Insurance (URBMI) were established, covering the majority of urban employees, urban residents and rural residents.

With the deepening of anti-poverty work, the dispersion of poor people and the increasingly complex causes of poverty, the past poverty alleviation methods are no longer suitable for the reality of poverty alleviation in China. Therefore, in 2013, President Xi Jinping proposed the concept of “targeted poverty alleviation” [4]. In response to this, in terms of medical insurance schemes, the catastrophic medical insurance (CMI) established in 2012 is also developing in depth, providing further protection for vulnerable people who are prone to poverty due to illness on the basis of basic medical insurance, and further deepening the bottom-line protection function of medical insurance for the poor. In 2018, it was mentioned in the Sixth National Health Services Statistical Survey Report that China's basic medical insurance coverage rate reached 96.8% [5, 6]. By the end of 2020, the basic medical insurance fund had financed a total of 230 million enrolments of the poor, and various medical insurance policies to help the poor had benefited a total of 530 million medical consultations, helping to reduce the medical burden by more than ¥360 billion and helping nearly 10 million households escape poverty precisely due to illness [7]. Both the medical insurance schemes and poverty alleviation policy at this stage have achieved population-wide coverage and the system's universal goal.

At the late stage of the task of eliminating absolute poverty, developing a strategy to further exert the poverty alleviation function of the medical insurance schemes has become an important agenda for targeted poverty alleviation. Moreover, in 2018, the proportion of China's poor households with disabilities and poverty caused by diseases exceeded 40% and 14%, respectively, and the proportion of people over 65 years old exceeded 16% [8]. Meanwhile, as a complement to the basic medical insurance, the CMI relies on the surplus operation of the NRCMS and URBMI, and follows the same list of reimbursements. As total medical costs continue to rise, the strength of medical insurance reimbursement may decline [9–11].

The incidence of CHE and medical poverty in China ranges from 15 to 45% [12]. Some studies suggest that it is largely related to the increased level of health service utilization and the absence of more generous reimbursement policies for outpatient care and medications as the most common health service needs and utilization items, especially for vulnerable populations with high health service needs and utilization and high out-of-pocket (OOP) cost levels, such as middle-aged and older populations [6, 13, 14]. A two-phase evaluation of China's healthcare reform by Professor Winnie Yip and others found that benefit packages such as the zero-markup drug policy and the revised fee schedule have not reduced total medical care expenditures, and irrational drug use continues [15]. In China, the incidence and mortality from chronic noncommunicable diseases are increasing due to the ageing population. In a national survey of 17 000 participants in 2017, 44.7% of the middle-aged and older population had hypertension [16]. The 2018 big data indicated that the utilization of basic public health services for the older was high, with the percentage of hypertensive and diabetic patients aged 60 and over who had received a health follow-up within a year both above 70% [3]. In 2019, deaths due to chronic diseases accounted for 88.5% of total deaths in China, including cardiovascular and cerebrovascular diseases and cancer. The proportion of deaths from chronic respiratory disease was 80.7% [17].

Based on this, we ask the following questions: Do middle-aged and older adults still have a high incidence of CHE as a high-need and high-health-utilization vulnerability population? In light of this marginal risk and the vulnerable group of people who suffer from poverty due to diseases, how do we precisely determine the role of the medical insurance scheme in the economic protection of this group? For these queries, we conducted a relevant study through the CHARLS data for related studies.

We use CHE, which is used as a measure of economic risk protection for universal health coverage, to measure the economic protection of the sample households [18–20]. Basic health insurance coverage, an economic risk-protective indicator for achieving universal health coverage in China, has negative utility association with the incidence of CHE. With the development of almost full coverage of basic medical insurance today, the consolidation of medical insurance schemes can provide the Chinese with even better economic protection of their health. Meng et al. [21], in a study published in 2015, already reached this rather forward-looking conclusion. China launched the urban and rural integrated medical insurance (URRMI) for urban and rural residents in 2016 following CMI in response to the high incidence of CHE. Initially, the URRMI was under the joint jurisdiction of the health department under the NRCMS and the social security department under the URBMI. It was then placed under the management of the newly established National Health Insurance Agency [22]. Based on the 2018 data, we will also explore the utility of URRMI for reducing the incidence of CHE.

## Methods

### Data source

This was a retrospective study, and mainly used the 2018 China Health and Retirement Longitudinal Study (CHARLS), which is a survey based on the Health and Retirement Survey (HRS). The survey subjects were randomly selected household heads aged 45 and above. The survey covered personal information, family members, health status, medical insurance status, income, expenditure and assets status. In addition, the CHARLS survey covered 450 villages and residences in 150 counties and districts across the country, including 17 708 individuals from 10 257 households, and generally represented the middle-aged and older adults in China.

### Unit of analysis and population and sample

This study first analyses regional differences in CHE across provinces and then further identifies the characteristics of middle-aged and older populations vulnerable to CHE on a household basis. In terms of data cleaning, based on the individual codes in the database, a code ending with “01” indicated the head of household. By integrating the household information of the householder, a household information database with the head of the household as the core and the household as the smallest unit formed a sample, and this study initially included a total of 9207 household samples. Samples containing missing values were further cleaned to finally obtain a sample database consisting of 9190 samples.

### Conceptual framework

Based on a literature research, we divided the influencing factors of CHE into three dimensions, namely sociodemographic characteristics, health service and utilization factors, and social security factors. Adisa found that older households with higher incomes and larger household sizes were less likely to suffer from CHE [23]. The incidence of CHE is relatively high in smaller households with high healthcare demands [24]. The demand and utilization of health services directly affects the health expenditure of residents, which in turn has an impact on whether CHE occurs [25]. Medical insurance is an important tool for alleviating poverty due to illness. Whether the head of the household is insured and the type of insurance will have an impact on CHE [26].

#### Calculation of CHE

Determining whether a household has incurred poverty, and CHE, is based on the methodology recommended by WHO. The indicators involved in the calculation include the following: total expenditure, which is the sum of all consumption expenditures of the household; subsistence expenditure, which is the expenditure on daily living costs; OOP, which is the expenditure on health-related services received by the household through OOP payment; capacity to pay (CTP), which is the non-subsistence expenditure component of total expenditure; and household health expenditure burden (OOP/CTP), if the proportion of household OOP to the CTP is greater than or equal to 40%, which is considered as indicative of the occurrence of CHE [27].

#### Selection of variables

Based on bibliometrics, we identified explanatory variables for the study as follows: (1) sociodemographic factors, including the householder’s age, marital status, education level, economic level (ranked according to the per capita household expenditure of the sample households and divided into five equal groups) and household size; (2) health service demand and utilization factors, including whether the householder had been an outpatient in the past month and whether the householder used inpatient services in the past year, whether households had disabled members, whether there was an older person aged 65 or above in the household, and whether the head of household had cardiovascular disease, chronic respiratory disease, cancer or diabetes; (3) social security factors, namely the type of householder's insurance, including UEBMI, URBMI, NRCMS, URRMI, and other insurance or no insurance. (For a comparison of the main basic medical insurance systems in China, see Additional file 1 for details, and for the specific source of independent variables, please refer to Additional file 2).

### Model selection

In the initial stage of the study, we focused on the endogeneity of the medical insurance system and tried to use “provincial participation rate” as an endogenous variable and “self-rated health” and “urban and rural” as instrumental variables. However, the results of the overidentification test rejected the null hypothesis that all instrumental variables were exogenous (*p* < 0.01) and indicated that our instrumental variables were null and related to the disturbance term. At the same time, we also tried to find other instrument variables to replace. Unfortunately, however, no valid instrumental variables were found based on our data. Logistic regression was eventually chosen to analyse the multidimensional risk factors affecting CHE. In Table3, the Hosmer–Lemeshow test was used to judge the model fit effect (*χ*^2^ (8) = 2.34, *P* = 0.96), which showed that the model had high goodness of fit.

## Results

### Basic information

The incidence of CHE increased with age and was highest in people aged 75 years and older (28.99%). The incidence of CHE tended to decrease with an increase in education level and household size, with both households with people over the age of 65 and those with disabilities having a higher incidence of CHE than households without such characteristics by about 10%. According to the National Bureau of Statistics, the provinces where the households were located were divided into eastern, central, and western regions, and the distribution of sample households and the prevalence of CHE were similar in the three regions (Table 1).

**Table 1:** Basic characteristics of householder and the incidence of CHE

	Value	Number (%)	Incidence of CHE(%)
Age of householder	1 = < 45	103 (1.1)	3.88
	2 = [45, 55)	2 488 (27.0)	11.94
	3 = [55, 65)	2 912 (31.6)	16.79
	4 = [65, 75)	2 404 (26.1)	25.37
	5 = ≥ 75	1 283 (13.9)	28.99
Education level of householder	1 = Illiterate	2 120 (23.0)	25.47
	2 = Primary School	3 239 (42.7)	20.49
	3 = Junior high school and above	3 141 (34.1)	13.59
Householder’s marital status	0 = Deceased spouse (including unmarried)	1 641 (17.8)	20.05
	1 = Spouse living (including divorced)	7 549 (82.1)	19.12
Whether the householder is insured	0 = No	287 (3.1)	13.24
	1 = Yes	8 903 (96.8)	19.48
Household size	1 = 1 person	1 075 (11.6)	22.70
	2 = 2–3 persons	7 718 (83.9)	19.14
	3 = > 3 persons	397 (4.3)	12.85
Whether there are individuals 65 years old and above in the household	0 = No	5 123 (55.7)	13.80
	1 = Yes	4 067 (44.2)	26.19
Whether the household member has a disability	0 = No	7 383 (80.3)	17.47
	1 = Yes	1 807 (19.6)	26.67
Whether poverty occurs in the household	0 = No	7 868 (85.6)	18.84
	1 = Yes	1 322 (14.3)	21.94
Household by region	1 = East	3 155 (34.3)	19.40
	2 = Central	3 032 (32.9)	18.50
	3 = West	3 003 (32.6)	19.95
Total	/	9 190 (100.00)	19.28

Value of composition ratio (%) ± 0.01

### Contrasting poverty rate, health expenditure burden and the incidence of CHE among different provinces

**Fig. 1:**
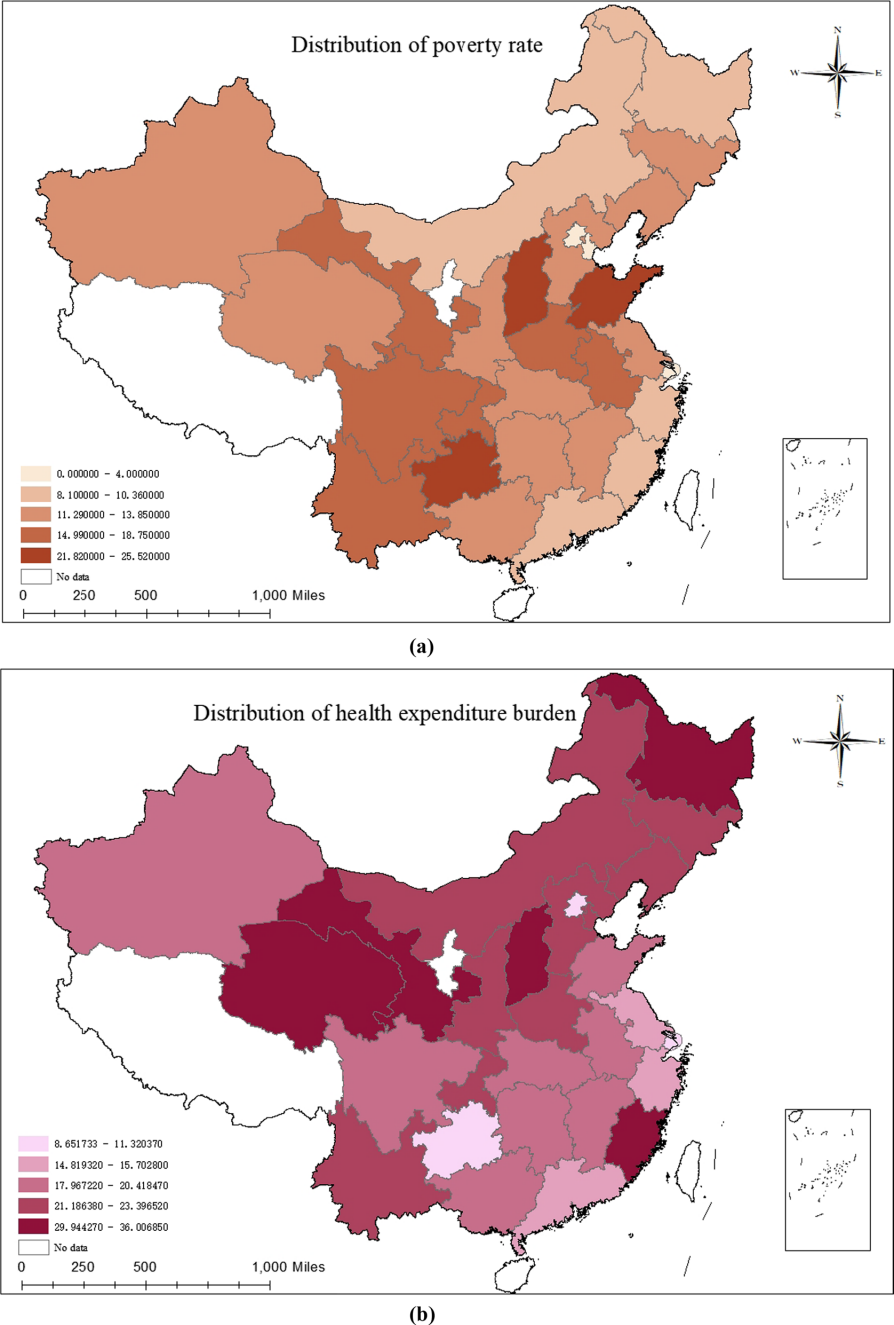

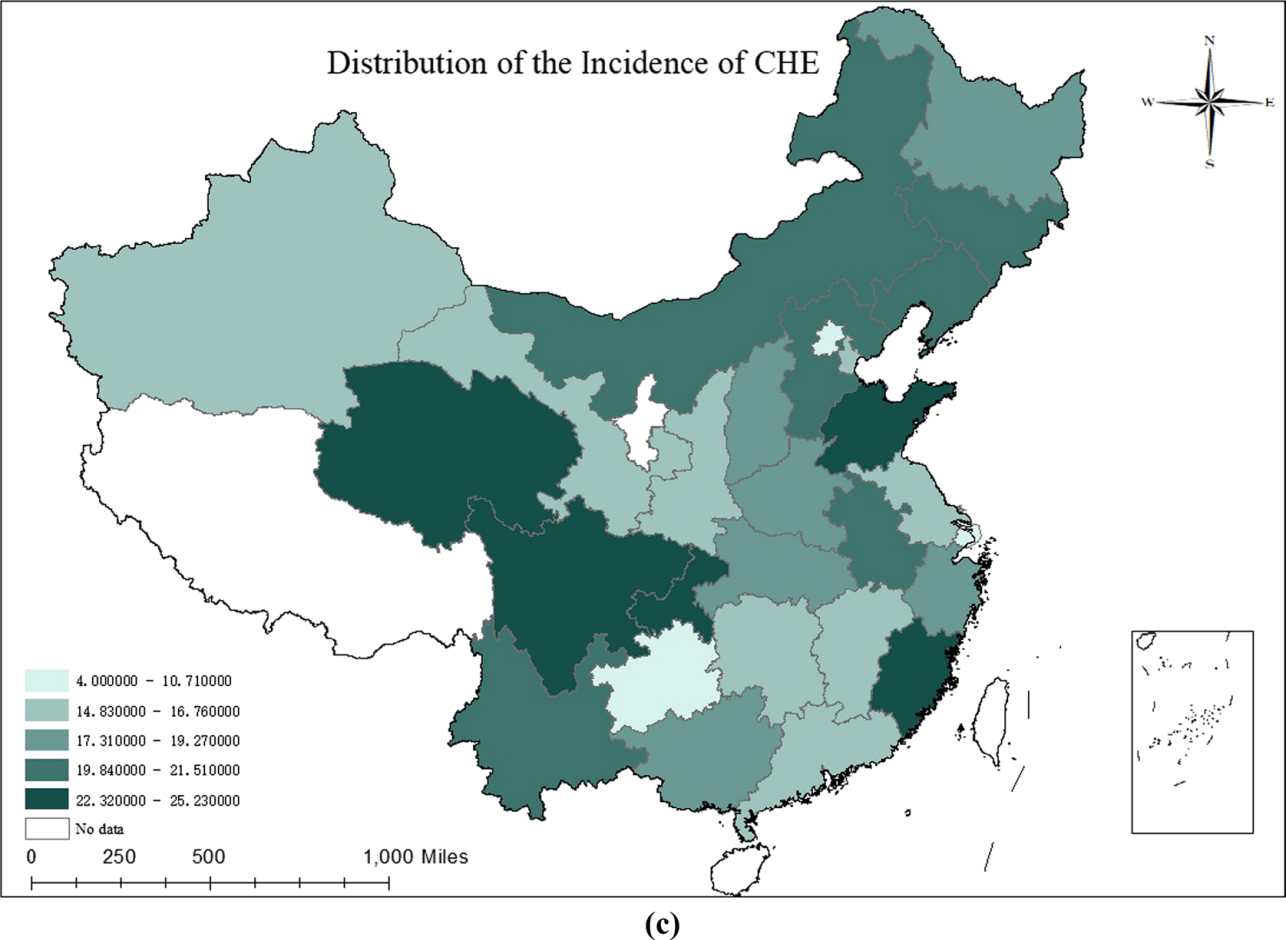
Regional distribution of poverty rate, health expenditure burden and the incidence of CHE

The poverty rate, health expenditure burden and CHE incidence were divided into five categories based on the natural breaks and are visualized in Fig. 1, where the darker the region, the larger the value, meaning the higher the incidence.

First, a longitudinal overview of the distribution of values among regions is presented. The province with the highest poverty rate was Shandong (25.52%), followed by Guizhou (24.35%), Shanxi (21.82%), Shanghai (4.00%), Tianjin (2.70%) and Beijing (0.00%). Qinghai had the highest health expenditure burden (36.01%), more than three times that of Shanghai, with the others in Fujian, Gansu and Shanxi all having burden above 30%. The highest CHE incidence was in Fujian (25.23%), followed by Sichuan (24.06%), which was more than six times the lowest CHE incidence in Shanghai.

In this study, the data results for Shandong, Yunnan and Chongqing provinces confirmed the same high trend in the three indicators of regional poverty rates, health expenditure burden and CHE incidence, and the data results for Guangdong, Beijing and Shanghai showed the same low trend. However, upon continuing horizontal comparison, we found something unusual. Using the poverty rate level as a baseline, Shandong and Guizhou had the highest poverty rates, with a small difference (1.17%), while their CHE incidence rates differed significantly (15.11% difference). Gansu Province, which had a high level of poverty and health expenditure burden, had a low incidence of CHE, while Yunnan, Sichuan and Chongqing, which were at the same level of poverty, had a higher incidence of CHE, with the maximum difference reaching almost 8.5%. Gansu, Fujian and Inner Mongolia shared a lower level of poverty, and had both relatively high health expenditure burden and high CHE incidence; in contrast, Guangdong, Zhejiang and Heilongjiang were located at the same low level of poverty, but the burden of health expenditure and the incidence of CHE were at a lower level (Fig. 1).

### Health expenditure burden and CHE incidence among households with different characteristics

The highest incidence of CHE was found in the poorest households (21.30%). Among households with a vulnerable characteristic population of zero to two people aged 65 years and above or persons with disabilities, the health expenditure burden and the incidence of CHE tended to increase with the number of persons with these characteristics. It can be seen that health expenditure burden and the incidence of CHE are higher in the diseased population than in the non-diseased population. The maximum difference in the health expenditure burden and the incidence of CHE were over 10% for both the population without chronic diseases and people with two or more chronic diseases. In terms of health service utilization, the health expenditure burden and the incidence of CHE were higher for people who had an outpatient experience in the past month and an inpatient experience in the past year (Table2).

**Table 2:** Health expenditure burden and CHE incidence among households with different characteristics

		OOP (yuan)	CTP (yuan)	Health expenditure burden (%)	Incidence of CHE (%)
Household economic level	Poorest	113.20	489.32	23.13	21.30
	Sub-poverty	251.23	1 071.53	23.45	20.94
	General	367.13	1 827.85	20.09	17.12
	Sub-rich	649.91	3 297.26	19.71	16.39
	Richest	2 203.49	10 710.16	20.57	20.67
Number of elderly people aged 65 and above in the household	0	657.05	4 036.86	16.28	13.80
	1 person	675.17	2 747.19	24.58	21.75
	2 persons	920.28	2 807.15	32.78	30.70
Number of persons with disabilities in the household	0	648.13	3 526.92	18.38	17.47
	1 person	1 010.77	3 343.36	30.23	25.61
	2 persons	984.64	2 847.34	34.58	34.58
Cardiovascular disease	No	683.59	3 422.47	19.97	18.03
	Yes	987.86	3 915.44	25.23	28.90
Chronic respiratory diseases	No	700.32	3 490.71	20.06	18.59
	Yes	973.04	3 321.17	29.30	28.87
Cancer	No	675.29	3 424.75	19.72	18.96
	Yes	3 903.01	7 498.19	52.05	43.09
Diabetes	No	709.06	3 450.79	20.55	18.93
	Yes	899.00	4 007.65	22.43	25.74
Number of chronic diseases	0	601.20	3 333.03	18.04	15.81
	1	697.83	3 495.99	19.96	21.95
	≥ 2	1 130.36	3 924.20	28.81	26.22
Outpatient in the past month	No	587.73	3 287.12	17.88	16.56
	Yes	1 360.96	4 421.29	30.78	32.63
Hospitalized in the past year	No	582.30	3 390.35	17.18	15.28
	Yes	1 392.35	3 918.24	35.54	39.02

*OOP* out-of-pocket health expenditure, *CTP* household's capacity to pay

### Comparison of overall health expenditure burden and CHE occurrence under different medical insurance types

**Fig. 2:**
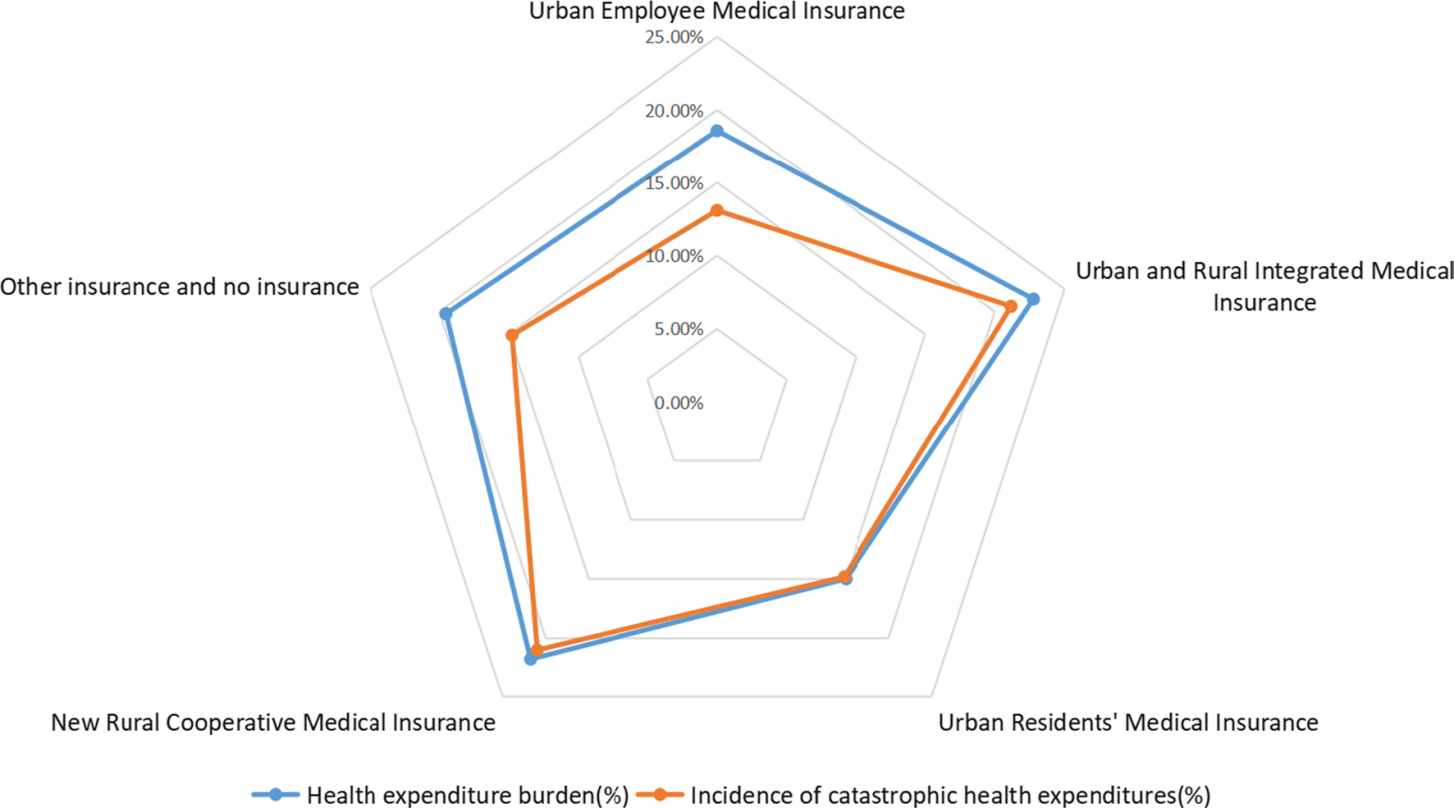
Overall health expenditure burden and incidence of CHE under different medical insurance types

**Fig. 3:**
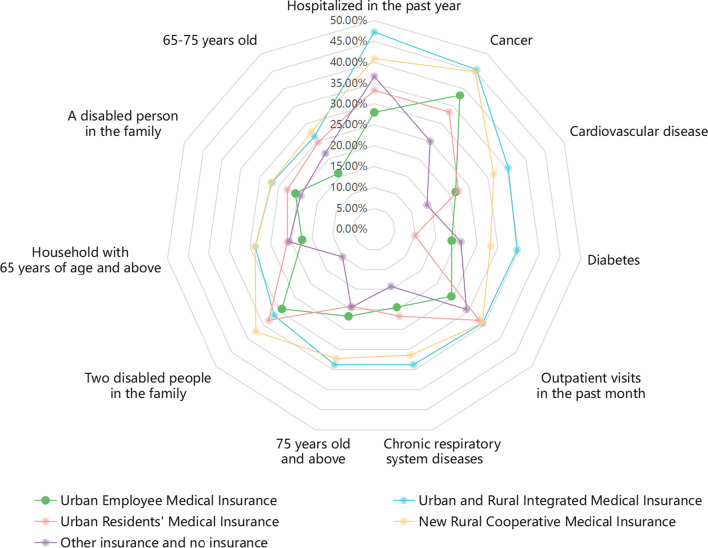
Incidence of CHE for each indicator under different medical insurance types

By calculating the health expenditure burden and the incidence of CHE for the population insured under the five different medical insurance schemes separately, we found that the health expenditure burden (22.77%) and the incidence of CHE (21.17%) were the highest for the group insured under the URRMI. Figure 2 presents a clearer view of the health expenditure burden and the incidence of CHE under different medical insurance schemes, and the top six indicators of the incidence of CHE under each type of medical insurance were selected to compare the incidence of CHE for the same indicator under different medical insurance schemes (Fig. 3). The incidence of CHE in the same characteristic population differs among different medical insurance schemes; for example, the incidence of CHE for the population with a household consisting of two disabled persons who are insured with other types of insurance or uninsured is shown with nodes pointing in the direction of low values, while the same characteristic population under the four types of medical insurance are curved towards high values.

### Regression results

We describe the results separately according to the data type. Results for the non-categorical variables are as follows: (1) Suffering from two chronic noncommunicable diseases including cardiovascular disease and cancer was positively correlated with the incidence of CHE, with cancer having a greater impact on the incidence of CHE. (2) Household characteristic factors including living spouse (including divorced), increasing age of the householder, household members with disabilities and household members over 65 years of age all aggravated the risk of CHE in the middle-aged and older groups. (3) In terms of health service utilization, the probability of occurrence of CHE in households whose head of household had used inpatient services in the past year was 2.80 times (95% confidence interval [CI] 2.456–3.192) that in households who had not used it. Similarly, the use of outpatient services in the past month was a significant factor in CHE. Households whose heads had used outpatient services were 2.24 times (95% CI 1.957–2.550) as likely to develop CHE as those who had not used outpatient services. For the categorical variables, the following was found: (1) Using the URRMI as the reference group, the risk of CHE occurrence was decreased in UEBMI, URBMI and those with other insurance or uninsured. (2) Compared to one-person households, the greater the household size, the lower the risk of CHE. (3) Using the poorest level as a reference, the risk of CHE occurrence decreased in the sub-rich household economic level. (4) In terms of education level, those with junior high school education and above had a lower risk of CHE than those who were illiterate (Table3).

**Table 3:** Results of regression

Variable		*B*	OR	95% CI of OR	*P* value
Medical insurance for householder	URRMI	Ref.			
	UEBMI	−0.695	0.499	0.393–0.633	0.000***
	URBMI	−0.455	0.634	0.459–0.877	0.006**
	Other insurance or no insurance	−0.515	0.598	0.439–0.813	0.001***
	NRCMS	−0.059	0.943	0.801–1.110	0.481
Householder with or without cardiovascular disease		0.242	1.274	1.056–1.536	0.011**
Householder with or without chronic respiratory diseases		0.094	1.099	0.878–1.376	0.410
Householder with or without cancer		0.729	2.073	1.384–3.106	0.000***
Householder with or without diabetes		0.181	1.199	0.933–1.540	0.155
Householder’s marital status		0.768	2.156	1.772–2.625	0.000***
Householder suffers from multiple chronic diseases		−0.006	0.994	0.927–1.065	0.866
Age of householder		0.029	1.030	1.020–1.039	0.000***
Whether the household member has a disability		0.284	1.329	1.166–1.514	0.000***
Household size	1 person	Ref.			
	2–3 persons	−0.417	0.659	0.535–0.813	0.000***
	> 3 persons	−0.568	0.567	0.392–0.820	0.003***
Whether there are individuals 65 years old and older in the household		0.225	1.252	1.041–1.506	0.017**
Whether householder has been an outpatient in the past month		0.804	2.234	1.957–2.550	0.000***
Whether householder has been hospitalized in the past year		1.030	2.800	2.456–3.192	0.000***
Education level of householder	Illiterate	Ref.			
	Primary school	−0.101	0.904	0.788–1.037	0.149
	Junior high school and above	−0.308	0.735	0.621–0.870	0.000***
Household economic level	Poorest	Ref.			
	Sub-poverty	0.019	1.019	0.861–1.206	0.827
	General	−0.139	0.870	0.729–1.039	0.124
	Sub-rich	−0.210	0.811	0.675–0.974	0.025**
	Richest	0.116	1.123	0.937–1.344	0.209
Cons_		−4.558	0.010		0.000
Model validation				Chi-square	*P* value
Hosmer-Lemeshow test				2.342	0.96

*OR* odds ratio, *CI* confidence interval

**p < 0.05, ***p < 0.01

## Discussion

Big data show that China has rapidly advanced on the path of poverty reduction since the reform and opening in 1978, with poverty alleviation contributing to more than 70% of global poverty reduction in the same period [28]. However, nearly 20% of middle-aged and older households in our study were still trapped by CHE; therefore, our research targeted the critical characteristics that would make these populations susceptible to CHE by analysing 2018 CHARLS data.

### Regional level: after China eliminates overall poverty, the risk of CHE continues to vary across regions

In general, regional poverty rates, health expenditure burden and CHE incidence all follow the same trend of being equally high or low. We found that the incidence of poverty and CHE were extremely similar in the east (14.48%), central (14.48%) and west (14.19%) (Table 1). Based on the results of data from Guizhou, Inner Mongolia, Heilongjiang and Fujian provinces [29], we concluded that the incidence of CHE in provinces does not necessarily follow the trend of their poverty incidence, which means that interregional macroeconomic development is not the main force in reducing the regional incidence of CHE. For middle-aged and older households, provincial poverty levels were not linked to their health expenditure burden, and high health service demand and utilization were the underlying causal factors for high CHE occurrence. The results of other studies showing a positively skewed relationship between CHE and medical care expenditure as a share of gross domestic product (GDP) corroborate our findings [12, 30]. More specifically, the provinces of Gansu, Shaanxi and Tianjin had a high level of medical care expenditure burden while maintaining a low incidence of CHE, an idiosyncratic performance that may be related to regional differences in medical subsidy policies. These findings show that region-based division should not be based solely on regional economic development level. Therefore, we should consider the differences in vulnerability among provinces, and each provincial government should take targeted action adapted to its provincial characteristics [19].

Provincial-level medical insurance funds should be coordinated, and the central government should adjust the flow of medical insurance funds across provinces. For example, economically developed regions with low CHE incidence may have large medical insurance fund balances, which will be handed over to the central government. Provinces with less developed economies or losses in medical insurance funds can upload their revenues and expenditures and budgets through the information-sharing platform, and the central government or other provinces will review them before allocating medical insurance funds.

### The level of social factors: the social vulnerability of middle-aged and elderly groups easily increases the risk of CHE

Our study showed that the older the household head, the greater the probability of CHE. In another study, the proportion of the population with multiple physical morbidities was shown to increase with age [31], and rising exposure to chronic noncommunicable diseases led to increasing demand for and utilization of medical services in middle-aged and older adults [6]. It is predicted that increasingly severe conditions associated with ageing will make it more difficult for the middle-aged and older adults to take advantage of the limited resources within their low capacity [32, 33]. This makes it more difficult for them to afford the cost of medical services, and as a result, the extremely high economic burden of disease ultimately leads to the occurrence of CHE in the middle-aged and older population [34].

Compared with living alone, the likelihood of CHE was 0.659 (95% CI 0.535–0.813) and 0.567(95% CI 0.392–0.820) times lower for households with 2–3 or more people living together, and the findings of other studies have shown that health expenditures and medical service demands were higher among married couples [35, 36]. Comparing the results based on household size in our research, the incidence of CHE was relatively high in smaller households with high healthcare demands [24]. For spouses alone, those with living spouses had doubly high medical service demands. However, they probably cannot afford to pay the double cost for medical services, leading to high CHE risk. At the household level, support from multiple members within large households can spread out the financial burden of medical care, thus effectively reducing the risk of household CHE. However, another study found that the occurrence of CHE was greatly increased in households with five or more members [37].

Additionally, in terms of household economic level, the regression results for sub-rich households showed protective effects against CHE at a significant level [38]. Compared with the poorest families, the risk of CHE decreased by 0.811 times (95% CI 0.675–0.974) in the sub-rich families. Compared to households at lower economic levels, households at higher economic levels had increased prevalence of multiple diseases and were more likely to have access to more or higher-quality medical services [31]. Wang and Chen et al. found that the rich used more medical services than the poor, but the expenditure reduction for the rich group was statistically significant compared to the poorest group, which means that the higher economic groups were able to receive larger reimbursement subsidies from insurance than the poor [39, 40].

### The overall basic medical insurance system level: the basic medical insurance scheme has limited protection for middle-aged and older adults with high demand and utilization of medical services and significant physiological fragility characteristics

Research has found that households falling under two categories of noncommunicable diseases, namely cardiovascular disease and cancer, are vulnerable to CHE, especially those with cancer patients [41, 42]. According to our regression results, the risk of CHE was 2.073 times (95% CI 1.384–3.106) higher in households where the head of household had cancer than in those without cancer. Although the association between the factor “multiple chronic diseases of the householder” and the incidence of CHE was not significant in our results, Zhao et al. [25] found that multiple diseases led to an increased likelihood of CHE. Moreover, in addition to the chronic disease group, groups with high medical service demands and utilization also had a high risk for CHE occurrence under all four types of medical insurance except for other insurance and no insurance (Fig. 3). The disabled and older adults over 65 years of age are hardly able to be providers of income and may even need additional care; thus, the households they belong to suffer heavier financial burden in the absence of labour, which leads to a high risk of CHE for themselves and their families [43, 44].

Based on existing research, the effectiveness of medical insurance for outpatient OOP has not been highlighted, especially in cases of increasing outpatient drug expenses, such that patients can only receive greater reimbursement through hospitalization [45]. Although medical insurance can substantially reduce drug expenditures for inpatient OOP, with total medical costs and OOP increasing rapidly, the reimbursement of non-pharmaceutical expenditures and sufficient discounts for outpatient drugs are lacking, and the risk of CHE is not effectively reduced for the insured population [14].

To develop a sustainable medical insurance scheme, we need to further explore the benefits coverage and compensation level of medical insurance. Using the results of this research, we hope to break through the limitations of existing medical insurance by addressing deep problems in URRMI and its process of development.

With increasing age, middle-aged and older people are exposed to a variety of physical, psychological and social characteristics of vulnerability, and are more prone to chronic diseases. It is inevitable that their demand for and utilization of health services will be high. On the one hand, we should strengthen the construction of primary medical institutions and implement a system of general practitioners and family doctors. Specifically, we should promote residents' medical demand for immersion into the community, strengthen the management of middle-aged and elderly patients with chronic diseases in communities, and provide all-around tracking care for middle-aged and older patients. In the daily management of chronic diseases, in addition to distributing free drugs to residents, we should improve tracking of residents' use of drugs and reminding them to take drugs correctly. Furthermore, we should expand the scope of chronic disease outpatient planning, increase the proportion of chronic disease outpatient hospitalization expenses and drug reimbursement, and expand the coverage of drug reimbursement.

### From URRMI alone: at present, URRMI is limited to mergers, and it still lacks preferential protection for the middle-aged and older vulnerable groups, and cannot achieve “1 + 1 > 2”

The benefit coverage of basic medical insurance is relatively inadequate for the health economic protection of the poor and medically vulnerable middle-aged and older population. Li et al. [46] found that the incidence of CHE for the total Chinese population was 13.0% in 2018. The incidence of household CHE in the middle-aged and older insured population in our study (19.48%) exceeded the incidence of CHE in the total population by 6.48%. The population of insured URRMI had a high burden of health expenditures (22.77%) and incidence of CHE (21.17%). The burden of health expenditures was relatively low among the population with other forms of insurance or no insurance. The low utilization of medical services of these households may explain the low incidence of CHE (Fig. 2). Huang et al. found that the increase in reimbursement rates for inpatient medical expenses of URRMI had a positive impact on medical service utilization among middle-aged and older rural residents, especially in the rural poor areas. At the same time, URRMI had a limited effect on hypertension, abnormal heart rhythm and obesity [47].

Furthermore, we found that the incidence of CHE in the chronic patient population who participated in URRMI was higher than that in other types of medical insurance. This may be because URRMI was newly established in 2017 and is still in the process of development [48]. First, the NRCMS and URBMI are still in the integration stage. Under the current trend of payment first before seeing a doctor, the difference between the two systems and the sudden expansion of the scope of reimbursement may lead to a delay in reimbursement, thus leading to a risk of CHE. Second, there are fundamental differences between urban and rural areas in terms of economic level and medical resources. Although the URRMI follows the principle of “high level of reimbursement, wide range of medication, and more choice of hospitals” to lower the threshold of medical service utilization, it does not provide targeted benefits to vulnerable populations [49]. In addition, increasing outpatient utilization and high drug demand among patients with chronic disease are the main reasons for their high disease economic burden [50]. Even though the URRMI has expanded the reimbursement drug catalogue, the high medical service utilization driven by high inpatient reimbursement rates, which results in high medical costs, may instead lead to CHE among patients with chronic diseases [46].

### Policy implementation: building multi-departmental coordination of poverty alleviation and medical insurance

The construction and popularization of policies takes time to accumulate. China's current medical insurance system has achieved coverage of more than 95% of the population, the function of universal benefit and sufficient coverage of the population. In the post-poverty era, the needs and risks of middle-aged and elderly people who are physically, psychologically and socially vulnerable are prominent. For such stubbornly poor and vulnerable groups, it is even more important to expand the medical insurance system and implement more effective protection for these groups.

First, in terms of the poverty alleviation system, regional poverty alleviation guided by regional economic levels does not precisely identify the risk of CHE occurrence for multi-vulnerable populations. China has already conducted targeted poverty alleviation through poverty registration and grassroots support. However, at the macro policy level, the government needs to considerable locoregional differences and enact corresponding countermeasures to alleviate poverty by taking into account the combined poverty level and occurrence of CHE in each province. In addition, multidimensional poverty alleviation needs to be carried out for middle-aged and older groups who are vulnerable in terms of economic income, social mobility, the need to be taken care of and so on, by integrating the efforts of multiple departments. For example, the China Disabled Persons' Federation and the Ministry of Civil Affairs provide services as a bundle to help the disabled in registering and applying for disability certificates. At the same time, the poverty alleviation office records the specific degree of poverty and disability, and then the Ministry of Human Resources and Social Security provides appropriate employment opportunities or skills training.

Second, medical insurance agencies are now dominated by the government, and as representatives of the demand side of medical services, they do not have the initiative to protect the insured against the economic risks of health [51–54]. This passivity will lead to inefficient reimbursement and delayed payment of compensation, thus exacerbating the risk of CHE under the high level of health service utilization of middle-aged and elderly people with multiple vulnerability characteristics.

**Fig. 4:**
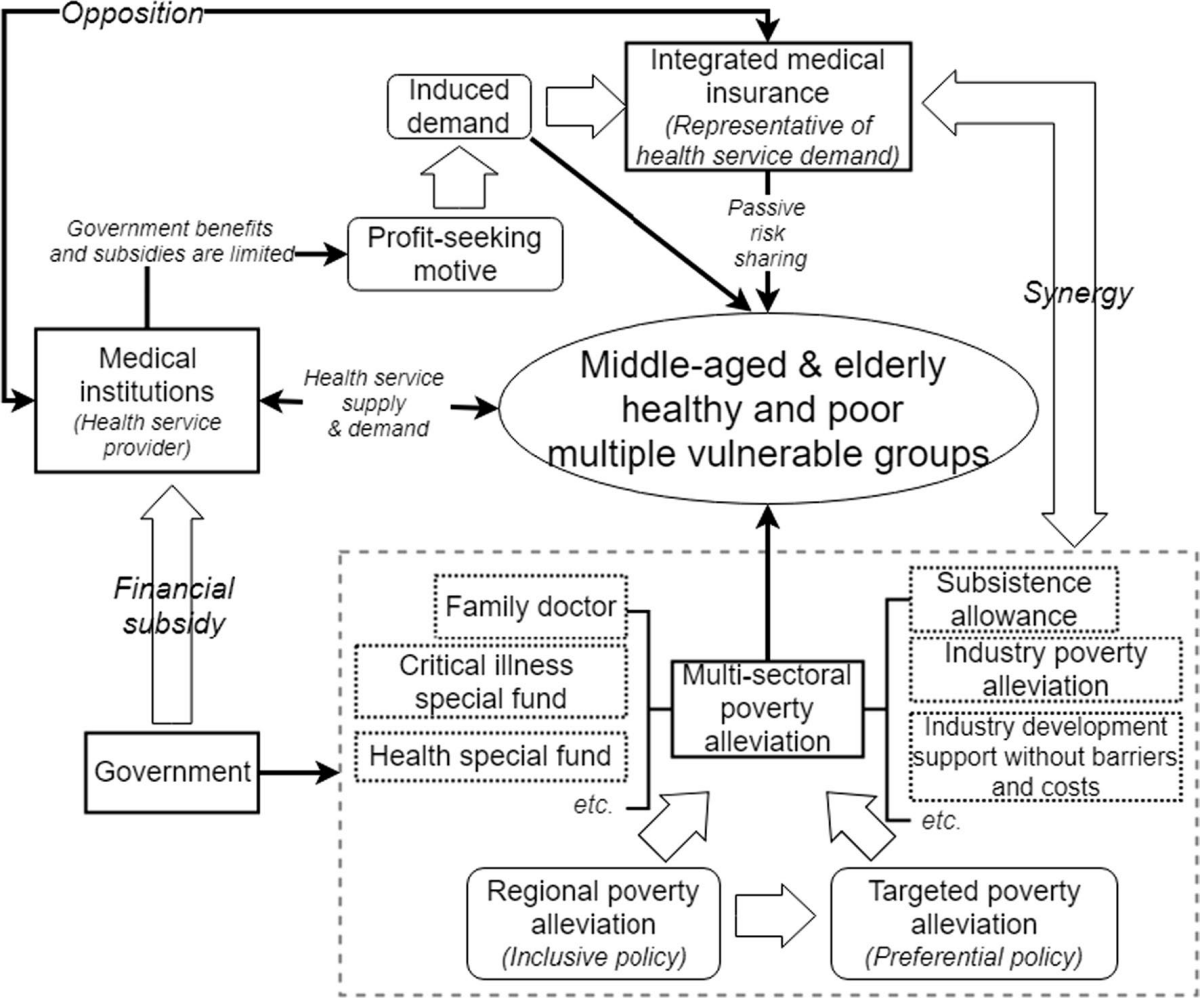
Synergistic relationship between poverty alleviation policies and medical insurance

Finally, medical insurance can reduce the cost of medical services for the middle-aged and older vulnerable groups to a certain extent; however, limited reimbursement and passive risk-sharing characteristics may cause a decline in the patients' quality of life after seeking medical care. Poverty alleviation policies such as family doctor, critical illness and health special funds exist, but factors such as their standard fees and reimbursement and conditions for application and use are still targeted towards the entire poor population without any special focus on the middle-aged and older adults who are vulnerable to health poverty. This may also be a concern for the uninsured and groups with low medical service utilization. Therefore, the poverty alleviation system can proactively provide physical and developmental sustainability for vulnerable groups among the poor and provide multifaceted support which targets vulnerability characteristics of the middle-aged and older adults. Between poverty alleviation policies and medical insurance schemes, the goal should be to strengthen the system of identifying and supporting vulnerable people so as to achieve an inclination towards “point-to-point” precise welfare (Fig. 4).

### Limitations and future perspectives

Some limitations exist in our research. First, to focus on the occurrence of CHE in the middle-aged and older population in 2018, we only performed a cross-sectional study. Our study lacks coherence in retracing emergent issues due to the development of medical insurance. Secondly, whether the lack of initiative of medical care providers leads to a decrease in the actual benefit experienced by participants should be the focus of follow-up studies. Additionally, CHARLS is a retrospective study. Respondents are often impressed by recent events and may have excessive feedback, which in turn may lead to selection bias and recall bias. This is also an unavoidable problem of retrospective research. In data cleaning, the core index calculated by CHE was used as the cleaning standard for observation variables. In order to retain the core index, other indicators may be omitted in data cleaning, such as household registration and family residence. This is also unavoidable in order to ensure the calculation of core indicators.

Next, we will use panel data to study the temporal evolution of CHE, and its multidimensional risk factors. Second, we can also use a spatial perspective in the future to focus on the heterogeneity of CHE occurrence between regions and implement appropriate interventions from the top of the region.

## Conclusion

With this study, we shed light on the characteristics of middle-aged and older adults and their households which make them vulnerable to CHE. The most important conclusion is that the level of macro-regional development is no longer a major protective factor in reducing the incidence of CHE in households. And the URRMI, whose utility is supposed to be the strongest, is instead more likely to have CHE in its insured group. If we can simultaneously prevent and solve poverty due to CHE, and achieve multisystem and multisectoral poverty alleviation for multiple vulnerable middle-aged and older adults, we can sooner achieve “poverty eradication” and “solve relative poverty”.

## Supplementary Information

**Additional file 1. **Comparison of major basic medical insurance systems in China.

**Additional file 2. **Main source of independent variables.

## Data Availability

The datasets generated during and/or analysed during the current study are available in the CHARLS repository [http://charls.pku.edu.cn/pages/data/111/zh-cn.html].

## Abbreviations

CHE: Catastrophic health expenditure
CHARLS: China Health and Retirement Longitudinal Study
CMI: Catastrophic medical insurance
URRMI: Urban and rural integrated medical insurance
NRCMS: New Rural Cooperative Medical Scheme
UEBMI: Urban Employee Basic Medical Insurance
URBMI: Urban Resident Basic Medical Insurance
OOP: Out-of-pocket
CTP: Capacity to pay
