# Supplementary material for: Degree of protection provided by poverty alleviation policies for the middle-aged and older in China: evaluation of effectiveness of medical insurance system tools and vulnerable target recognition
Source: Health Res Policy Syst. 2022 Nov 14;20:129. doi: 10.1186/s12961-022-00929-9 (PMC9664814; doi:10.1186/s12961-022-00929-9)
Supplement: Supplementary file 1 — Additional file 1. Comparison of major basic medical insurance systems in China. [file 12961_2022_929_MOESM1_ESM.docx]

Additional file 1 Comparison of major basic medical insurance systems in China

| Medical insurance schemes | Applicable groups | Range of medical benefits | Financing systems |
| --- | --- | --- | --- |
| Urban employee basic medical insurance (UEBMI) | All urban employing units, including enterprises ,institutions, public institutions, social organisations, private non-enterprise units and their employees. | Due to the differences in regional economic development level, medical insurance development level and disease spectrum, the inclusion of chronic and special diseases in medical insurance outpatient clinics varies among provinces. The number of chronic diseases included in the UEBMI outpatient clinics in various provinces in China is ranked as hypertension, chronic kidney disease, diabetes, mental illness, tuberculosis, aplastic anemia, liver cirrhosis, lupus erythematosus, and malignant tumor. | Medical insurance contributions are paid by both the employer and the individual, with the employer's contribution rate controlled at around 6% of the employee's gross salary and the employee's contribution rate generally at 2% of his or her salary income. |
| Urban resident basic medical insurance (URBMI) | Urban minors and non-working residents who do not participate in urban employees' medical insurance. | The order of the number of chronic diseases included in the URBMI outpatient clinics in each province in China is hypertension, chronic kidney disease, mental illness, diabetes, tuberculosis, and lupus erythematosus. | The funding of URBMI is mainly based on a combination of individual contributions and financial subsidies, with financial subsidies tilted towards people in need. |
| New rural cooperative medical schemes (NRCMS) | Chinese rural residents without other medical insurance | The diseases co-ordinated by the NRCMS outpatient clinics mainly include hypertension, coronary heart disease, diabetes, sequelae of acute cerebrovascular accident, and severe mental illness. | The NRCMS adopts the approach of individual contribution, collective support and government funding to raise funds. |
| Rural residents basic medical insurance (URRBMI) | The coverage of the URRBMI includes all the existing URBMI and the NRCMS participants. | The order of the number of chronic diseases included in the URRBMI outpatient clinics in each province in China is hypertension, chronic kidney disease, mental illness, diabetes, tuberculosis, and lupus erythematosus. | A combination of individual contributions and government subsidies is the main funding method. |
| Catastrophic medical insurance (CMI) | Major medical insurance covers the URBMI and the NRCMS participants. | Medical expenses that are not covered by the URBMI and the NRCMS, and further protection for high medical expenses incurred by patients with catastrophic diseases. | Individual contributions and a certain percentage or amount from the URRBMI are used to fund CMI. |
