# Supplementary material for: Degree of protection provided by poverty alleviation policies for the middle-aged and older in China: evaluation of effectiveness of medical insurance system tools and vulnerable target recognition
Source: Health Res Policy Syst. 2022 Nov 14;20:129. doi: 10.1186/s12961-022-00929-9 (PMC9664814; doi:10.1186/s12961-022-00929-9)
Supplement: Supplementary file 2 — Additional file 2. Main source of independent variables. [file 12961_2022_929_MOESM2_ESM.docx]

Additional file 2 Main source of independent variables

| Independent variables | Source |
| --- | --- |
| Age of householder | Sajad Vahedi et al.(2020) |
|  | Boyoung Jeon,Haruko Noguchi et al.(2017) |
|  | Evandro F.Fan,Chenglong Xie et al.(2020) |
| Education level of householder | Yi Zhang et al.(2019) |
|  | Anup Karan et al.(2017) |
|  | Yuqi Ta et al.(2020) |
| Householder’s marital status | Yang Zhao et al.(2020) |
|  | Miaoqing Yang(2018) |
|  | Xian Huang,Bingxiao Wu(2020) |
| Economic level | Ankit Viramgami et al.(2020) |
|  | Felicien Ilunga-Ilungaet al.(2015) |
|  | Mark W Moses MHSet al.(2019) |
| Provincial participation rate | Anwen Zhang et al.(2017) |
|  | Xuezhu Shi(2020) |
|  | Diana Cheung,Ysaline Padieu (2015) |
| Medical insurance for householder | Juliet S.Okoroh et al.(2020) |
|  | Jingshan Chen,De-Cai Tian et al.(2020) |
|  | Qiangyue Meng et al.(2015) |
| Householder suffers from multiple chronic diseases | Xia-Tong Ke et al.(2021) |
|  | L Ye,B.-C.Shia et al.(2019) |
|  | Bidisha Mondal,Jay Dev Dubey (2020) |
| Whether the household member has a disability | Sajad Vahedi et al.(2020) |
|  | Boyoung Jeon,Haruko Noguchi et al.(2017) |
|  | L Ye,B.-C.Shia et al.(2019) |
| Whether householder has been outpatient in the past month | Yang Zhao et al.(2020) |
|  | Taehwan Park,Moncia Hwang(2020) |
|  | Mark W Moses MHSet al.(2019) |
